# Beyond Time Loss: Position-Specific Injury-to-Performance Pathways After Hand Fractures in Elite European Football

**DOI:** 10.7759/cureus.117345

**Published:** 2026-10-02

**Authors:** Eduardo Campos Martins, Pedro L Paredes Argotte

**Affiliations:** 1 Institute of Orthopedics and Traumatology, Universidade de São Paulo, São Paulo, BRA

**Keywords:** athlete rehab, bundesliga, football, goalkeeper, hand fracture, hand surgeon, major league soccer, premier league, sports physiotherapy, traumatic injury

## Abstract

Background: Hand fractures are common sports injuries, but the same diagnosis may carry different performance consequences according to how essential the hand is to a player's role. We investigated whether fracture burden and the broader injury-to-performance pathway differed by playing position, from injury phenotype and acute absence to competitive reintegration and sustained participation.

Materials and methods: This retrospective matched cohort study linked manually validated public injury reports with league appearances from the five major leagues, Premier League, LaLiga, Serie A, Bundesliga, and Ligue 1 from 2007/08 through 2024/25. Of 35,952 injury records, 329 candidate records with possible hand or wrist injuries (0.9%) were reviewed, from which 148 fracture events (45.0% of candidates) in 142 players were retained. Primary analyses compared incidence, time loss, competitive return, and one-year participation after the publicly reported injury end. Exploratory analyses tested position-specific fracture phenotype and the time period from the public injury-end milestone to first subsequent appearance in one of the five major leagues.

Results: Fracture phenotype differed by position (chi-square = 21.48, P < .001; Cramér's V = 0.38): finger/phalangeal fractures accounted for 31 of 63 goalkeeper events (49.2%) versus 15 of 85 outfield events (17.6%) (odds ratio (OR), 4.52; 95% confidence interval (CI), 2.15-9.52), whereas metacarpal fractures accounted for 10 of 63 goalkeeper events (15.9%) versus 34 of 85 outfield events (40.0%) (OR, 0.28; 95% CI, 0.13-0.63). Goalkeepers faced greater time loss than outfielders, even within the same fracture subtype (position-by-subtype interaction, P = .407). Median time loss was 43 days among 63 goalkeeper events versus 18 days among 85 outfield events (adjusted ratio, 2.10; 95% CI, 1.58-2.79). By 90 days, 17 of 45 goalkeepers (37.8%) versus 55 of 68 outfield players (80.9%) had reappeared competitively. Of 103 fracture episodes with an observed appearance after the reported injury end, 14 of 35 goalkeeper episodes (40.0%) versus seven of 68 outfield episodes (10.3%) required more than 90 additional days to reappear (aOR, 6.94; 95% CI, 2.06-23.41; false discovery rate-adjusted q = .005). In the matched cohort, a 30% or greater participation decline occurred in 10 of 18 goalkeeper fracture cases (55.6%) versus 21 of 72 goalkeeper controls (29.2%) (aOR, 3.30; 95% CI, 1.08-10.08), with a fracture-by-goalkeeper interaction OR of 5.74 (95% CI, 1.46-22.66); this interaction estimate was based on a small subgroup of 18 goalkeeper fracture cases.

Conclusions: The important positional signal extends beyond the already expected higher fracture incidence in goalkeepers. Playing position was associated with differences in fracture phenotype, acute burden, the transition from the publicly reported injury-end milestone to subsequent competitive selection, and subsequent participation. Return-to-play counseling should therefore distinguish the publicly reported injury-end milestone from subsequent competitive reintegration and incorporate role-specific hand function rather than calendar time alone.

## Introduction

Hand fractures are common in sport and demand a balance between stable healing, preservation of tendon gliding and motion, and timely return to activity [1-5]. The available return-to-sport literature is generally reassuring: many athletes return quickly, particularly after stable injuries or fixation that permits early motion. However, systematic reviews have emphasized heterogeneous cohorts, inconsistent rehabilitation criteria, and limited evidence about the quality and durability of return [3,6]. A calendar date therefore answers only part of the clinical question.

Published experience also tends to pool sports or positions in which the injured hand can be protected, taped, splinted, or temporarily removed from the defining action. American football and collegiate cohorts frequently report return within one to four weeks, and selected professional contact athletes have returned approximately two weeks after stable fixation [7-10]. Baseball and basketball studies similarly report high return rates and limited average performance deterioration after metacarpal fracture [11,12]. These findings are useful, but their transferability depends on whether the athlete can function while the hand is still protected or incompletely restored.

Association football or soccer creates a particularly informative contrast because players share the same competitive environment while the occupational importance of the hand differs sharply by position. Outfield players primarily run, kick, and contest the ball with the lower extremities and can often remain eligible with a protected hand. On the other hand, goalkeepers repeatedly catch, parry, punch, grip, absorb high-velocity impact, and land on the hand. Prospective European surveillance has already shown that goalkeepers sustain markedly more hand, wrist, and forearm injuries and longer absences than outfield players [13,14], while long-term clinical observations describe greater finger and wrist symptoms in experienced goalkeepers [15]. Thus, the higher acute burden in goalkeepers is not itself surprising.

The unresolved question is what happens after that obvious incidence difference. Return to sport is a continuum rather than a single event, integrating tissue recovery, sport-specific function, confidence, training exposure, selection, and restoration of the prior competitive role [16]. We therefore examined whether the injury-to-performance pathway differed by playing position after a publicly reported hand fracture. In addition to incidence and acute time loss, we evaluated fracture phenotype, first competitive reappearance, the interval between the publicly reported injury-end milestone and subsequent selection, and one-year participation relative to same-position matched controls. We hypothesized that the positional divergence would extend beyond acute absence and would remain detectable after accounting for fracture subtype.

## Materials and methods

Study design and data sources

This retrospective matched cohort study linked publicly available injury histories from Transfermarkt (https://www.transfermarkt.com/) with player profiles and league appearances in the five major leagues (Big Five): the men's Premier League, LaLiga, Serie A, Bundesliga, and Ligue 1 from the 2007/08 through 2024/25 seasons. The reporting of this study followed the STROBE (Strengthening the Reporting of Observational Studies in Epidemiology) statement and sport-injury reporting principles adapted to a public-data setting [17-20].

The study analyzed only publicly available information and did not access medical records, diagnostic imaging, or protected health information. Under local institutional policy, institutional review board approval and informed consent were not required. The source competitions were men's professional leagues; therefore, sex was not analyzed as a comparative variable.

Case identification and manual validation

The broad phenotype included records whose public injury description was “metacarpal fracture,” “broken finger,” “broken thumb,” “hairline crack in middle finger,” or “broken hand.” The strict sensitivity phenotype retained only records with anatomically explicit metacarpal or phalangeal fracture labels and excluded the nonspecific "broken-hand" injuries. Wrist, scaphoid, forearm, dislocation-only, and soft-tissue labels were excluded from both phenotypes. 

Candidate hand, finger, thumb, wrist, and distal-forearm labels were identified from the free-text injury-reason field using a prespecified dictionary. Among 35,952 public injury records, 329 candidate records had possible hand, finger, thumb, wrist, or distal-forearm injury labels. All 329 records were manually reviewed against the anatomic and fracture definitions. Of these, 148 hand-fracture events in 142 players met the study criteria and were retained. The strict sensitivity phenotype retained only records with anatomically explicit metacarpal or phalangeal fracture labels and excluded the nonspecific “broken hand” category. It included 98 of the 148 validated fracture events.

Fractures were classified as metacarpal, finger/phalangeal, thumb, or unspecified hand fracture. The first qualifying fracture per player was used for incidence, competitive-return, and matched-trajectory analyses to limit disproportionate contribution from recurrent events; all validated fracture events contributed to descriptive and time-loss models. Playing position at the index season was classified as either "goalkeeper" or "outfield". 

Outcome framework

We assessed four complementary domains along the injury-to-performance pathway: player-season incidence; acute burden, measured as calendar days lost and matches missed; competitive return, defined as the first subsequent appearance in one of the five major leagues and including the interval from the publicly reported injury-end date to reappearance; and one-year participation/performance after the publicly reported injury end relative to same-position matched controls.

Acute burden comprised calendar days lost and matches missed. Player-season incidence was calculated for the 2012/13 through 2024/25 seasons, during which appearance-derived denominators were consistently observable, and expressed per 1,000 player-seasons. Competitive return was defined as the first subsequent appearance in one of the five major leagues after injury onset, with return probabilities estimated at 28, 42, 60, 90, 180, and 365 days.

The publicly reported injury-end date was treated as a data-source milestone rather than as confirmed union, full training, or medical clearance. Among fracture episodes with an observed post-end Big Five appearance, we measured the interval from the publicly reported injury-end milestone to the first subsequent appearance in one of the Big Five leagues. This analysis was intentionally interpreted as selection-ready competitive reintegration rather than biological healing.

Each qualifying public injury record from Transfermarkt was treated as one injury event, whereas incidence denominators were player-seasons with observable league participation [19]. For incidence analyses, the denominator was expressed in player-seasons: one player with observable participation in a Big Five league during a given season counted as one player-season [17].

Matched participation analysis

Players with a qualifying hand fracture were eligible for the matched analysis when they had accumulated at least 450 league minutes during the 365 days before injury and had complete pre-injury and post-episode observation windows. The first qualifying fracture per player was used for this analysis. Each player was matched to four same-position controls from the same season and competition, within three years of the player's age and selected for proximity in baseline minutes. Primary controls had no recorded hand or wrist injury during the 730 days preceding the index date. A separate sensitivity analysis used controls with no recorded injury of any type during that period.

Controls received the player's season-aligned index date and a pseudo injury-end date displaced by the same reported injury duration. The primary trajectory outcome was a decline of at least 30% in league minutes during the 365 days after the publicly reported injury end relative to the 365 days before injury. Secondary analyses used 20% and 50% thresholds, post-index rather than post-end windows, continuous post-to-pre minute ratios, market-value decline, exit from the five major leagues, and subsequent publicly reported hand or wrist injury. Pre-index placebo models examined whether cases were already diverging before the fracture.

Statistical analysis

Calendar days lost were modeled with gamma regression using a log link, and matches missed with negative binomial regression. Models were adjusted for age, fracture subtype, competition, season phase, prior injury burden, and repeated events within players. Median regression quantified the adjusted absolute positional difference. Poisson regression with player-season offsets estimated the goalkeeper-to-outfield incidence rate ratio. Time to first competitive reappearance was evaluated with Kaplan-Meier methods and Cox regression, and 90-day return was additionally modeled with logistic regression. Players were followed from injury onset until their first subsequent Big Five reappearance and were administratively right-censored at 365 days or, when earlier, at the end of observable Big Five follow-up. 

Matched-trajectory models included fracture exposure, goalkeeper position, the fracture-by-goalkeeper interaction, age, log-transformed baseline minutes, and competition indicators, with cluster-robust standard errors by matched set. Conditional logistic regression, strict fracture definitions, controls without any recorded injury, alternative participation thresholds, exclusion of pandemic-affected seasons, and pre-index placebo analyses were used to test robustness. Because goalkeeper cells were modest, Firth-penalized logistic regression with profile-likelihood confidence intervals (CIs) was used for the goalkeeper effect and the interaction; additional analyses restricted goalkeepers to those with at least 1,500 or 1,800 pre-injury minutes.

To determine whether the positional difference was more than a simple consequence of different fracture case mix, exploratory analyses compared subtype distributions with a chi-square test and Cramér's V, calculated subtype-specific positional odds ratios (ORs), and added a position-by-fracture-subtype interaction to the primary time-loss framework. Among fracture episodes with an observed post-end appearance, the goalkeeper and outfield distributions of the post-end reintegration lag were compared nonparametrically. Logistic models then estimated the adjusted ORs (aORs) of requiring more than 28, 60, or 90 days after the public injury-end milestone to reappear, adjusting for age, acute time loss, and fracture subtype with player-clustered standard errors. Benjamini-Hochberg false discovery rate correction was applied within related exploratory model families. Effect magnitude, precision, and consistency were emphasized over isolated P values. All analyses were performed using Python version 3.13.5 (Python Software Foundation, Wilmington, Delaware, United States), primarily with statsmodels version 0.14.6 and SciPy version 1.17.0; data management was performed using pandas version 2.2.3 and NumPy version 2.3.5.

Treatment strategy was not analyzed because the public injury feed did not reliably encode operative versus nonoperative management, fixation method, immobilization, protective equipment, laterality, hand dominance, imaging severity, or rehabilitation protocol. A field that did not label an event as surgery was not interpreted as evidence of nonoperative care.

## Results

Cohort and fracture phenotype

Of 35,952 public injury records, 329 hand/wrist injuries (0.9%) underwent manual review. The final broad cohort contained 148 fracture events (45.0%) in 142 unique players; this included 63 goalkeeper events (42.6%) and 85 outfield events (57.4%). The subsequent analytic cohorts were overlapping subsets or derived comparison cohorts and therefore should not be summed. Specifically, 98 of the 148 fracture events (66.2%) met the strict explicit metacarpal/phalangeal phenotype; 113 of the 142 players (79.6%) had observable competitive-return follow-up.

The matched participation analysis comprised 66 players drawn from the original fracture cohort plus 264 non-fracture same-position controls, yielding 330 matched observations. Thus, the study included 148 validated fracture events overall, not 541 fractures. Median age at the first qualifying fracture was 27.1 years in both groups.

These analyses used different, partly overlapping analytic samples rather than separate groups of participants. The strict phenotype included 98 of the 148 fracture events; competitive-return follow-up was available for 113 of 142 players; and the matched participation analysis included 66 fracture cases from the original cohort plus 264 matched controls. These counts refer to different analyses and should not be added together.

The study flow and the relationship among the analytic cohorts are shown in Figure 1.

**Figure 1:**
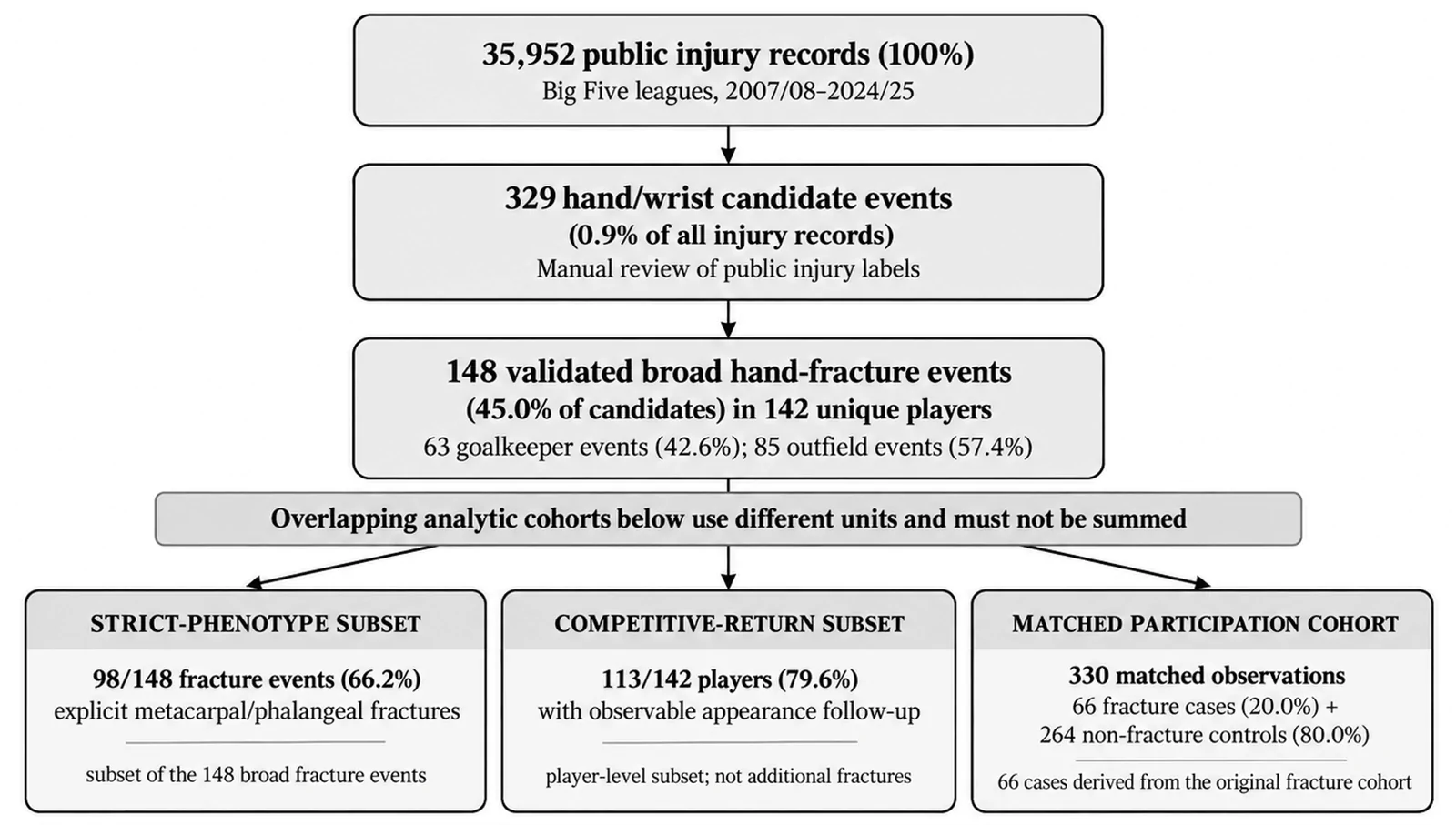
Study flow and overlapping analytic cohorts. Manual validation reduced 329 hand/wrist injury records to 148 validated broad hand fracture events in 142 unique players. The three lower boxes represent overlapping analytic subsets or derived comparison cohorts and are not additive. Of the 148 fracture events, 98 (66.2%) met the strict explicit metacarpal/phalangeal phenotype. Competitive return follow-up was available for 113 of 142 players (79.6%); this is a player-level subset of the original fracture cohort rather than an additional set of fracture events. The matched participation analysis included 66 fracture cases drawn from the original fracture cohort and 264 non-fracture same-position controls, yielding 330 matched observations. Accordingly, the total number of validated fracture events remained 148.

The distribution of publicly reported fracture phenotypes differed by playing position (chi-square = 21.48, P < .001; Cramér's V = 0.38). Finger/phalangeal fractures accounted for 31 of 63 goalkeeper events (49.2%) but 15 of 85 outfield events (17.6%), corresponding to a goalkeeper-versus-outfield OR of 4.52 (95% CI, 2.15-9.52; false discovery rate-adjusted q < .001). Metacarpal fractures showed the opposite pattern, accounting for 10 goalkeeper events (15.9%) and 34 outfield events (40.0%) (OR, 0.28; 95% CI, 0.13-0.63; q = .004). Thumb and unspecified-hand categories showed no clear independent positional difference (Table 1 and Figure 2).

**Table 1:** Cohort characteristics, fracture phenotype, and acute burden by position. The overall fracture-subtype distribution differed by playing position (chi-square = 21.48, P < .001; Cramér's V = 0.38). Percentages in the Events column are calculated using the 148 validated broad fracture events as the denominator, and percentages in the Players column use the 142 unique players as the denominator. Fracture-subtype percentages are calculated within each position using the corresponding number of fracture events as the denominator. IQR: interquartile range

Position	Events (n=148), n (%)	Players (n=142), n (%)	Age (years), median (IQR)	Metacarpal, n (%)	Finger/phalangeal, n (%)	Thumb, n (%)	Unspecified hand, n (%)	Days lost, median (IQR)	Matches missed, median (IQR)
Goalkeeper	63 (42.6%)	58 (40.8%)	27.1 (22.9-32.0)	10/63 (15.9%)	31/63 (49.2%)	5/63 (7.9%)	17/63 (27.0%)	43 (29-65)	7 (3.5-11)
Outfield	85 (57.4%)	84 (59.2%)	27.1 (23.4-29.6)	34/85 (40.0%)	15/85 (17.6%)	3/85 (3.5%)	33/85 (38.8%)	18 (11-30)	3 (1-5)

**Figure 2:**
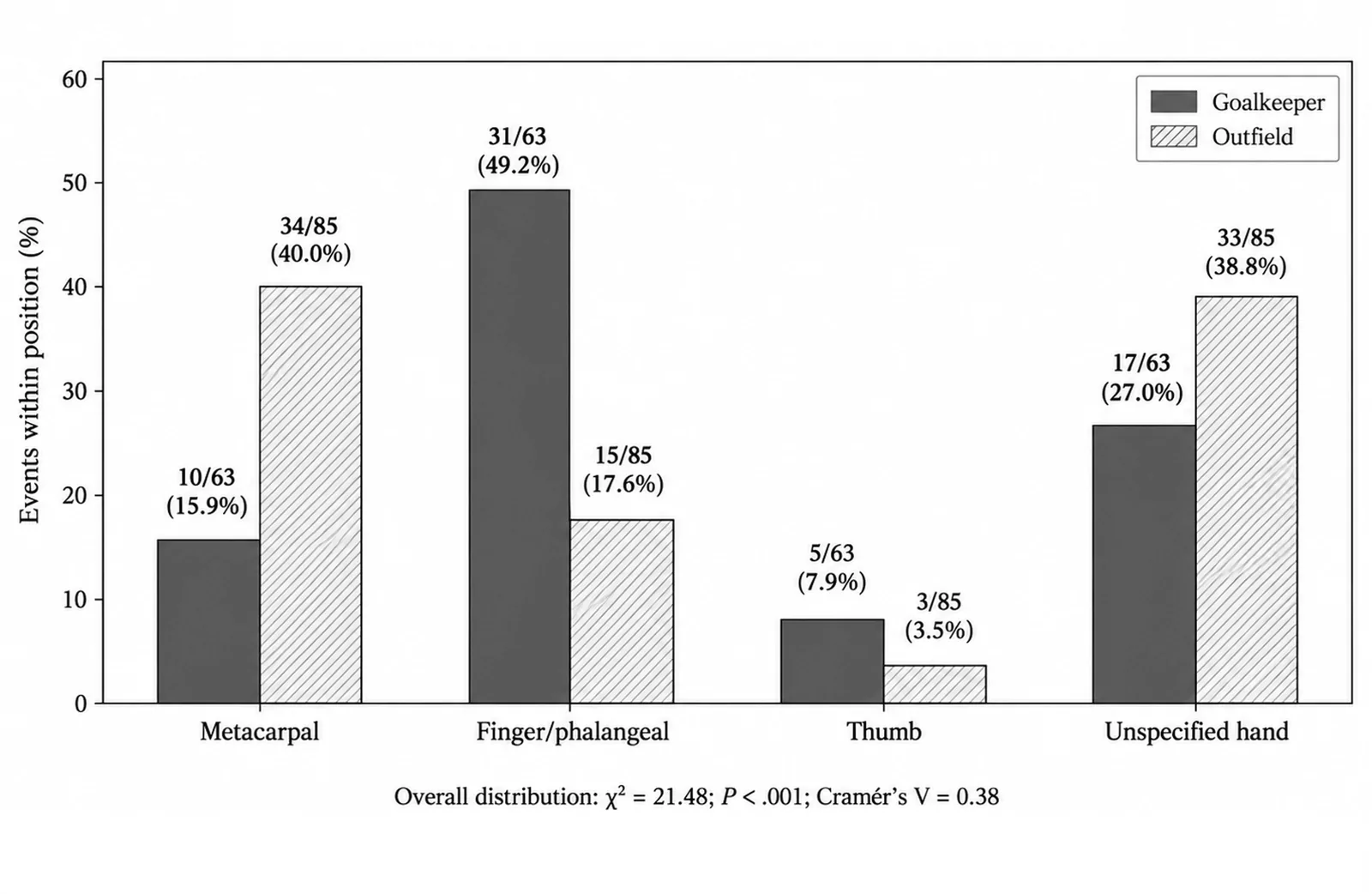
Fracture phenotype by playing position. The event-level distribution differed by position (chi-square = 21.48, P < .001; Cramér's V = 0.38). Goalkeepers were enriched for finger/phalangeal fractures, whereas metacarpal fractures were more common among outfield players.

Acute burden and the role of fracture subtype

Goalkeepers lost a median of 43 days (interquartile range (IQR), 29-65) and seven matches (IQR, 3.5-11), compared with 18 days (IQR, 11-30) and three matches (IQR, 1-5) among outfield players. From 2012/13 onward, incidence was 18.08 goalkeeper fractures and 2.14 outfield fractures per 1,000 player-seasons, yielding an adjusted incidence rate ratio of 8.38 (95% CI, 5.79-12.12). After adjustment, goalkeepers accumulated 2.10 times the calendar days lost (95% CI, 1.58-2.79), 2.48 times the matches missed (95% CI, 1.82-3.38), and 18.9 additional median days absent (95% CI, 10.7-27.1) (Table 2).

**Table 2:** Adjusted position-specific effects across the acute and competitive-return pathway. CI: confidence interval; HR: hazard ratio; IRR: incidence rate ratio; OR: odds ratio

Outcome	Adjusted comparison	Effect	95% CI	p value
Fracture incidence	Goalkeeper vs outfield	IRR 8.38	5.79-12.12	< .001
Calendar time loss	Goalkeeper vs outfield	Ratio 2.10	1.58-2.79	< .001
Matches missed	Goalkeeper vs outfield	Ratio 2.48	1.82-3.38	< .001
Median calendar time loss	Goalkeeper minus outfield	+18.9 days	10.7-27.1	< .001
Competitive return by 90 days	Goalkeeper vs outfield	OR 0.09	0.03-0.27	< .001
Time to competitive return	Outfield vs goalkeeper	HR 2.99	1.83-4.90	< .001

The different fracture mix did not explain the goalkeeper time-loss penalty. The position-by-subtype interaction was not significant (P = .407), while the adjusted goalkeeper-to-outfield time-loss ratio remained greater than one in each publicly reported fracture subtype: 2.96 (95% CI, 1.65-5.29) for metacarpal fractures, 1.59 (95% CI, 0.97-2.60) for finger/phalangeal fractures, 2.01 (95% CI, 1.01-4.03) for thumb fractures, and 2.17 (95% CI, 1.38-3.42) for unspecified hand fractures. Thus, the acute positional difference persisted within fracture phenotype rather than being attributable only to a different distribution of labels.

Competitive return and two observable time intervals

The competitive-return analysis included 113 of 142 players (79.6%) with observable appearance follow-up, comprising 45 of 58 goalkeepers (77.6%) and 68 of 84 outfield players (81.0%). By 28 days after injury onset, five of the 45 goalkeepers (11.1%) and 29 of the 68 outfield players (42.6%) had made a subsequent Big Five appearance. By 60 days, the corresponding numbers were 16 of 45 goalkeepers (35.6%) and 50 of 68 outfield players (73.5%); by 90 days, it was 17 of 45 (37.8%) and 55 of 68 (80.9%), respectively; and by 365 days, 29 of 45 (64.4%) and 64 of 68 (94.1%), respectively. Outfield players returned at nearly three times the instantaneous rate of goalkeepers (hazard ratio (HR), 2.99; 95% CI, 1.83-4.90), and the adjusted odds of a goalkeeper appearing within 90 days were 0.09 (95% CI, 0.03-0.27) relative to an outfield player.

The exploratory post-injury-end analysis separated the publicly reported injury episode from subsequent competitive selection. Among 103 fracture episodes in 100 players with an observed appearance after the publicly reported injury-end milestone, public injury duration was longer in goalkeepers (median 35 days, IQR 25.5-47.0) than outfield players (14.5 days, IQR 10.0-30.0). The subsequent lag from the public injury-end milestone to first Big Five appearance had a median of 15 days (IQR 8-176) for goalkeepers and 11 days (IQR 2-40.5) for outfield players (Mann-Whitney U test, P = .023). The more informative difference was in the long tail: 14 of 35 goalkeeper episodes (40.0%) versus 11 of 68 outfield episodes (16.2%) required more than 60 days after the public injury end to reappear, and 14 goalkeeper episodes (40.0%) versus seven outfield episodes (10.3%) required more than 90 days. After adjustment for age, acute time loss, and fracture subtype, goalkeeper episodes had 4.32-fold higher adjusted odds of a post-end lag greater than 60 days (95% CI, 1.47-12.69; q = .012) and 6.94-fold higher adjusted odds of a lag greater than 90 days (95% CI, 2.06-23.41; q = .005) (Table 3 and Figure 3).

**Table 3:** Exploratory decomposition of competitive reintegration after the publicly reported injury end. Adjusted prolonged-lag models included age, acute time loss, and fracture subtype with player-clustered standard errors. The unit of analysis was the fracture episode; 103 eligible episodes occurred in 100 unique players. This observed-returner analysis describes selection-ready competitive reintegration and is not a measure of confirmed medical clearance. FDR: false discovery rate; IQR: interquartile range; OR: odds ratio

Analysis	Goalkeeper	Outfield	Adjusted effect	95% CI	FDR q
Public injury duration, median (IQR)	35 d (25.5-47.0)	14.5 d (10.0-30.0)	Descriptive	Not applicable	Not applicable
Post-end lag, median (IQR)	15 d (8-176)	11 d (2-40.5)	Mann-Whitney U test; P = .023	Not applicable	Not applicable
Post-end lag >60 days	14/35 episodes (40.0%)	11/68 episodes (16.2%)	OR 4.32	1.47-12.69	.012
Post-end lag >90 days	14/35 episodes (40.0%)	7/68 episodes (10.3%)	OR 6.94	2.06-23.41	.005

**Figure 3:**
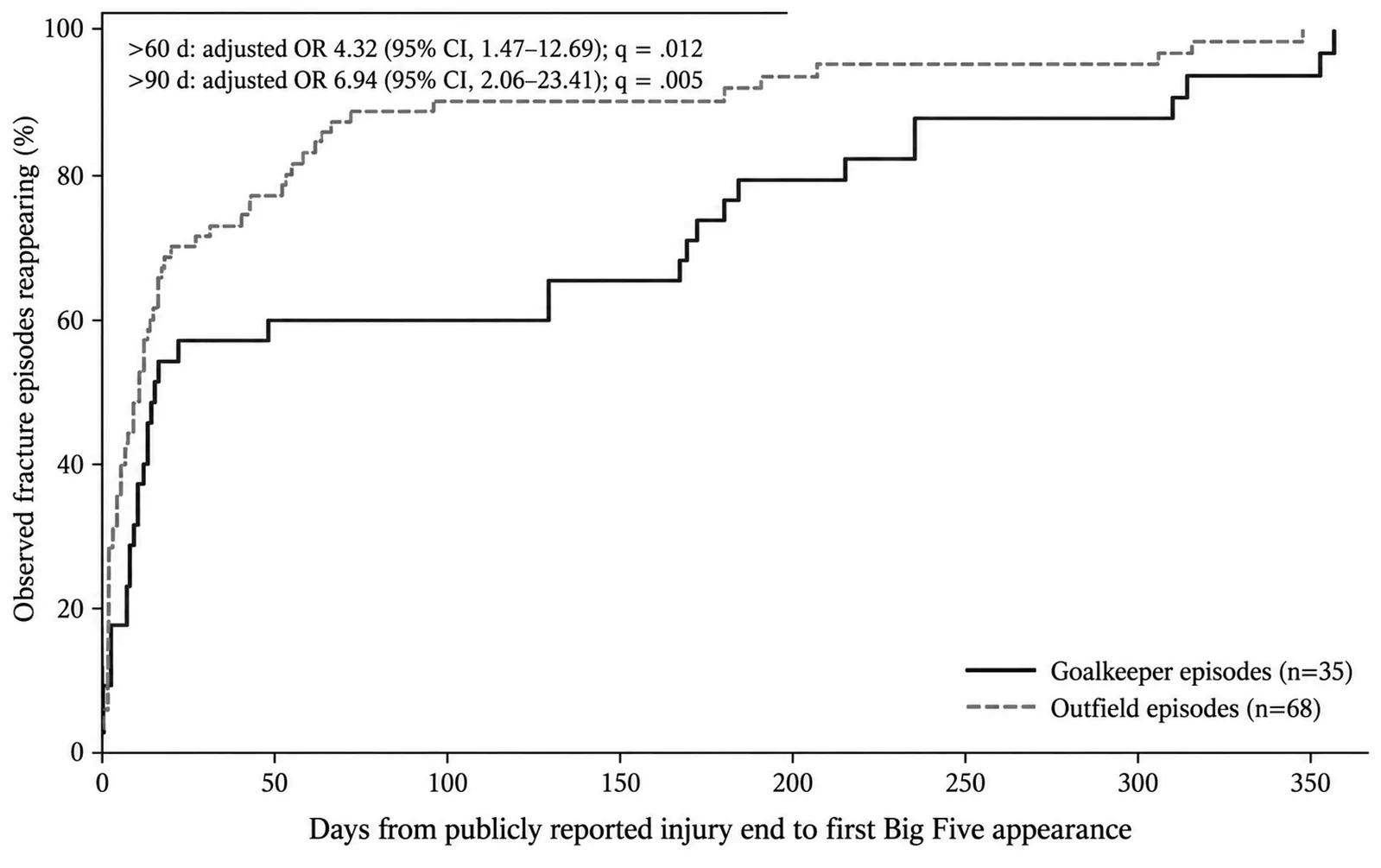
Competitive reintegration after the publicly reported injury episode ended. Empirical cumulative reappearance among fracture episodes with an observed post-end Big Five (men's Premier League, LaLiga, Serie A, Bundesliga, and Ligue 1) reappearance. The public injury-end date is a data-source milestone and is not equivalent to medical clearance. Adjusted odds of requiring more than 60 or 90 days to reappear were estimated after adjustment for age, acute time loss, and fracture subtype.

Matched cohort analysis

The primary matched cohort comprised 330 observations: 66 fracture cases (20.0%) and 264 same-position controls (80.0%). Among the fracture cases, 18 were goalkeepers (27.3%) and 48 were outfield players (72.7%); among controls, 72 were goalkeepers (27.3%) and 192 were outfield players (72.7%). Balance was close for age and baseline minutes. A decline of at least 30% in participation after the publicly reported injury-end milestone occurred in 10 of 18 goalkeeper fracture cases (55.6%) and 21 of 72 goalkeeper controls (29.2%), giving an aOR of 3.30 (95% CI, 1.08-10.08). Among outfield players, the corresponding rates were 14 of 48 fracture cases (29.2%) and 79 of 192 controls (41.1%) (OR, 0.57; 95% CI, 0.26-1.26). The fracture-by-goalkeeper interaction OR was 5.74 (95% CI, 1.46-22.66) (Table 4). Because the goalkeeper fracture subgroup comprised only 18 cases, these estimates were relatively imprecise despite reaching statistical significance and should be interpreted cautiously. Accordingly, the magnitude of the position-specific participation association should be considered hypothesis-generating and requires confirmation in larger cohorts.

**Table 4:** Matched participation after the publicly reported injury-end milestone. Fracture cases comprised 18 goalkeepers and 48 outfield players; Controls comprised 72 goalkeepers and 192 outfield players. The primary trajectory window began at the publicly reported injury-end date, not confirmed medical clearance. Firth penalized estimates were OR 3.15 (95% profile CI, 1.06-9.84) for the goalkeeper subgroup and OR 5.31 (95% profile CI, 1.51-19.35) for the fracture-by-goalkeeper interaction. CI, confidence interval; OR, odds ratio.

Analysis	Fracture rate, n (%)	Control rate, n (%)	Adjusted OR	95% CI	p value
Broad goalkeeper	10/18 (55.6%)	21/72 (29.2%)	3.30	1.08-10.08	.036
Broad outfield	14/48 (29.2%)	79/192 (41.1%)	0.57	0.26-1.26	.166
Broad fracture × goalkeeper	Not applicable	Not applicable	5.74	1.46-22.66	.013
Strict goalkeeper	8/13 (61.5%)	13/52 (25.0%)	5.16	1.19-22.43	.029
Strict outfield	12/33 (36.4%)	51/132 (38.6%)	0.79	0.31-2.01	.617
Strict fracture × goalkeeper	Not applicable	Not applicable	6.55	1.13-37.92	.036

Firth-penalized estimates were similar for the goalkeeper subgroup (OR 3.15, 95% profile CI, 1.06-9.84) and the interaction (OR 5.31, 95% profile CI, 1.51-19.35). The interaction persisted with the strict explicit phenotype and was directionally similar when controls with any recorded injury were excluded. Among established goalkeepers with at least 1,500 pre-injury minutes, the point estimate remained similar but became less precise (Firth OR 3.09, 95% profile CI, 0.84-11.81). Pre-index placebo, market-value, major-league exit, and subsequent hand/wrist injury analyses showed no clear independent adverse association, indicating that the dominant signal was concentrated in participation rather than demonstrable career termination or recurrent injury.

## Discussion

The most informative finding was not that goalkeepers sustain more hand fractures. That gradient is clinically intuitive and has already been demonstrated prospectively [13]. The principal observation is that playing position was associated with differences across the injury-to-performance pathway. The groups differed in fracture phenotype, goalkeepers remained absent longer even within the same public fracture subtype, competitive selection was slower, the long tail of reintegration persisted beyond the publicly reported injury-end milestone, and subsequent participation was reduced relative to same-position controls. A hand fracture in elite football is therefore not a position-neutral sporting exposure.

The phenotype analysis helps separate mechanism from consequence. Nearly half of goalkeeper events were finger/phalangeal fractures, compared with fewer than one-fifth of outfield events, whereas metacarpal fractures were more prominent among outfield players. This distribution is compatible with the repeated catching, parrying, punching, hyperextension, and direct ball-impact mechanisms intrinsic to goalkeeping [13-15]. Importantly, however, case mix was not the whole explanation. The absence of a meaningful position-by-subtype interaction and the persistence of goalkeeper time-loss ratios above one across all four fracture categories indicate that role-related demands remain relevant even after comparing similar public injury phenotypes.

This distinction matters because published return intervals are often transferred across sports as if fracture stability were the dominant determinant of readiness. Selected athletes can return early after fixation when stability, symptoms, protection, and the sport's rules permit it [7-10,21,22]. That evidence supports early motion and carefully protected participation, but it does not imply that all athletes with the same fracture should share the same competitive timetable. A goalkeeper cannot meaningfully spare the hand from catching, punching, high-velocity ball contact, glove-mediated grip, and landing during dives. Residual stiffness, swelling, altered digital convergence, tendon adhesion, contact sensitivity, or apprehension may therefore remain performance-limiting despite satisfactory biological healing [23-25].

We distinguished two observable intervals in the public dataset. The first spans injury onset to the publicly reported injury-end date; the second, measured only for episodes with an observed post-end appearance, spans that milestone to the first subsequent Big Five appearance. Neither interval represents a biological stage of recovery, confirmed healing, return to full training, or formal medical clearance. The median post-end lag differed only modestly, but the distributions diverged sharply in the tail: 14 of 35 goalkeeper episodes (40.0%) with an observed post-end appearance required more than 90 additional days, compared with seven of 68 outfield episodes (10.3%). This estimate remained large after adjustment for age, acute time loss, and fracture subtype. The public injury-end date cannot be equated with medical clearance, but that is precisely why the finding is useful: it shows that public-data episode closure and selection-ready performance represent distinct observable milestones, and the interval between them differed by position.

Goalkeeping may magnify this distinction because the competitive role is comparatively indivisible. An outfield player can return for limited minutes, enter late in a match, or perform with a protected hand while direct hand exposure is progressively restored. Goalkeeper substitution is uncommon, and selection usually requires immediate readiness for the complete role from the first minute. The critical difference may therefore be less that goalkeepers simply 'use their hands more' and more that their position offers little opportunity for graded competitive reintegration. A partially recovered goalkeeper often cannot be partially deployed.

The matched analysis extends this concept beyond the first appearance. High return rates after metacarpal fracture in professional baseball and basketball have often been accompanied by limited average performance deterioration [11,12,26]. Our outfield results were compatible with that favorable prognosis. Goalkeepers were different: the participation decline exceeded ordinary same-position volatility, and the formal fracture-by-position interaction persisted under stricter definitions and penalized estimation. At the same time, the lack of a clear signal for market-value decline, exit from the major leagues, or subsequent hand/wrist injury argues against an unnecessarily catastrophic interpretation. The dominant pattern was a concentrated disruption of competitive participation rather than inevitable career failure. Although the fracture-by-goalkeeper interaction remained statistically significant and was directionally consistent across the strict-phenotype and Firth-penalized analyses, the goalkeeper fracture subgroup was small (n=18) and the confidence intervals were wide. The consistency of the signal is therefore noteworthy, but its magnitude should be regarded as hypothesis-generating until replicated in larger cohorts.

These findings support a role-based framework for rehabilitation and return decisions. For a goalkeeper, radiographic stability, absence of pain at rest, or the first competitive reappearance should not be treated as equivalent to full occupational readiness. Progression should consider pain-free glove use, rapid grip formation and release, repeated catching and parrying, punching, ground contact, contested aerial balls, fatigue-dependent dexterity, apprehension, and the next-day symptom response. Existing hand-fracture rehabilitation literature emphasizes stability, motion, protection, and individualized return [4-6,22,25], while football-specific and collegiate studies show that injury management and time loss vary substantially with exposure context [27-29]. The present data add that the positional role itself should be part of prognostic counseling from the outset.

The study also defines what cannot be concluded from public data. Operative versus nonoperative management, fixation method, immobilization, protective equipment, hand dominance, radiographic severity, and rehabilitation protocol were not reliably available. Accordingly, the observed differences cannot be attributed to a specific treatment strategy, nor can a missing surgery label be interpreted as conservative management. The study is best understood as an analysis of the injury-to-performance pathway, not a comparative-effectiveness study of treatment.

Strengths and limitations

Strengths include nearly two decades of multi-league observation, manual validation of every candidate fracture label, position-specific denominators, separation of injury-end and competitive reappearance milestones, same-position matched controls, formal effect-modification testing, and Firth-penalized analyses for small goalkeeper cells.

Limitations include the absence of clinical imaging and examination, uncertainty about the exact fracture segment in nonspecific labels, potential undercapture of minor injuries that were never publicly reported, lack of athlete-exposure denominators, and unmeasured influences such as contract status, goalkeeper hierarchy, club return-to-play practice, transfers, and unrelated health events. Such undercapture may also be differential by playing position. Minor hand fractures in outfield players may be less likely to generate a publicly recorded injury episode when the athlete can continue or return while using protection, whereas comparable goalkeeper injuries may be more visible because unrestricted hand function is integral to the position. Differential ascertainment could therefore inflate the observed goalkeeper-to-outfield incidence gradient and may also distort comparisons of apparent injury burden. As the reporting threshold cannot be measured from public data, the direction and magnitude of this bias cannot be quantified.

Because fracture categories were derived from free-text public labels rather than imaging or medical records, some apparent positional differences in fracture phenotype may reflect variation in reporting specificity or terminology rather than true anatomic differences. The observed-returner post-end analysis is descriptive of selection-ready reintegration among fracture episodes with a recorded subsequent appearance; player clustering was used to account for repeat observations. It should not be interpreted as a causal estimate of medical clearance. Finally, the findings apply to men's elite European football and require confirmation in women's football, youth cohorts, and other hand-dependent sporting roles.

## Conclusions

The important positional signal extends beyond the expected higher incidence of hand fractures in goalkeepers. Playing position was associated with differences in fracture phenotype, acute burden, competitive return, the transition from the publicly reported injury-end milestone to subsequent selection, and one-year participation. Importantly, the excess goalkeeper time loss persisted within fracture subtype, indicating that the positional difference cannot be explained solely by a different distribution of fracture labels. The findings therefore support viewing a hand fracture not as a position-neutral diagnosis, but as an injury whose sporting consequences depend on the functional demands of the role to which the athlete must return.

Clinically, return after a hand fracture should be considered a staged process rather than a single date. The publicly reported injury-end milestone, first competitive reappearance, and restoration of sustained participation represent distinct milestones, particularly for goalkeepers, whose role provides limited opportunity for graded competitive reintegration. Return-to-play decisions should therefore integrate role-specific hand function, tolerance of repeated impact, glove use, grip performance, confidence, fatigue-dependent dexterity, and the ability to sustain the full positional workload rather than relying on calendar time alone. Prospective studies incorporating imaging, treatment strategy, objective hand-function testing, training exposure, and selection data are needed to determine which factors account for the prolonged competitive reintegration observed in goalkeepers.
